# Neoadjuvant Treatment Approaches to Oral Cancer

**DOI:** 10.3390/jcm14196883

**Published:** 2025-09-28

**Authors:** Lyna Siafa, Aisha Ali, Paul Kerr, Alok Pathak, Norbert Viallet, Ciaran Lane, Suhail Sayed

**Affiliations:** 1 Department of Otolaryngology—Head and Neck Surgery, University of Manitoba, Winnipeg, MB R3T 2N2, Canada; 2 Max Rady College of Medicine, University of Manitoba, Winnipeg, MB R3E 3P5, Canada; 3 Department of Surgery, University of Manitoba, Winnipeg, MB R3A 1R9, Canada

**Keywords:** oral squamous cell carcinoma (OSCC), neoadjuvant, immunotherapy, PD-1, PD-L1, CTLA4, chemotherapy

## Abstract

**Background/Objectives**: The high prevalence of oral squamous cell carcinoma (OSCC) has driven the development of surgical and oncologic techniques to improve survival. Despite advancements in surgical technique and chemoradiation protocols, survival rates for locally advanced OSCC remain low due to high recurrence and metastasis. This has driven the exploration of neoadjuvant treatment protocols as a potential pathway towards improving organ-preserving resection, de-escalating adjuvant treatment, and improving overall and recurrence-free survival. **Methods**: This is a narrative review summarizing the current literature and ongoing trials on neoadjuvant treatment for OSCC. PubMed was searched using a snowballing technique to capture all relevant clinical trials. **Results**: 21 clinical trials were identified. Although neoadjuvant chemotherapy was associated with favorable pathologic outcomes, clinical trials demonstrated variable survival outcomes. In contrast, neoadjuvant immunotherapy for OSCC demonstrated improved pathologic responses and survival outcomes, with a low incidence of grade 3–4 adverse events. **Conclusions**: Neoadjuvant therapy in OSCC shows promise but does not yet constitute standard of care. Neoadjuvant immunotherapy has encouraging response rates and lower treatment-related toxicities in comparison to neoadjuvant chemotherapy. Although recent clinical trials have presented strong evidence to support the use of neoadjuvant immunotherapy in the treatment of locally advanced OSCC, further randomized trials are required to establish standardized neoadjuvant protocols and biomarkers to assess treatment response.

## 1. Introduction

Oral squamous cell carcinoma (OSCC) is the most common form of head and neck cancer. Locoregionally advanced disease is conventionally treated using surgery with or without adjuvant radiotherapy or chemoradiotherapy based on the presence of high-risk pathologic features [1,2]. However, survival remains poor in locoregionally advanced disease due to high rates of recurrence and metastasis [1]. Concurrent systemic therapy and radiation therapy is often the treatment of choice for advanced disease but is also associated with treatment-related toxicities and mortality [3].

Despite advancements in surgical techniques, oncologic outcomes remain poor for OSCC, driving exploration of neoadjuvant strategies to improve survival outcomes [1]. Neoadjuvant chemotherapy has been explored in OSCC with the aim of reducing tumor burden, facilitating surgical resection, and preserving organ function [4,5]. Its clinical utility however remains a topic of debate, due to concerns regarding treatment-related toxicities, potential surgical delays, and inconsistent survival outcomes reported across trials [2,6].

Beyond chemotherapy, neoadjuvant immunotherapy has emerged as a promising alternative that leverages the patient’s immune system in the preoperative setting to debulk tumors [7]. By reducing tumor burden preoperatively, it may open the door to de-escalation of adjuvant treatment and prevention of further treatment-related complications [1]. Neoadjuvant immunotherapy may also have the benefit of influencing long-term disease control through immune-mediated mechanisms, though its full clinical utility remains under investigation in head and neck cancers [7].

Immune checkpoint inhibitors targeting the programmed cell death protein 1 (PD1) pathway and Cytotoxic T-lymphocyte-associated protein 4 (CTLA4) have emerged as the most commonly used biologics in neoadjuvant immunotherapy. By modulating immune tolerance in the tumor microenvironment, these agents can enhance the priming and activation of T cells [7]. A number of early-phase clinical trials have suggested that neoadjuvant immunotherapy has promising tolerability and pathologic response in locally advanced and recurrent OSCC, with potential survival benefits when compared to the current standard of care. This has resulted in a growing interest within current research on the integration of neoadjuvant immunotherapy within multimodal treatment frameworks to improve patient outcomes in OSCC [1,8]. Most recently, the KEYNOTE-689f clinical trial demonstrated improvements in event-free survival with neoadjuvant pembrolizumab in locally advanced head and neck squamous cell carcinoma (HNSCC) compared to the current standard of care (surgery and adjuvant chemoradiation) [9]. This phase III trial presents high-level evidence in support of neoadjuvant immunotherapy as a treatment for locally advanced OSCC [9].

This narrative review was conducted to synthesize key developments in neoadjuvant treatment strategies for OSCC, with a focus on emerging clinical evidence, investigational therapies, and ongoing research gaps. By mapping the current landscape, this review aims to outline future directions in the pursuit of risk-adapted care.

## 2. Materials and Methods

A narrative review was conducted. A PubMed search was performed from 1 July to 15 July 2025, using the terms ‘neoadjuvant’ and ‘oral cancer’. A snowballing technique was applied to key publications, reviewing their references to ensure inclusion of all landmark studies. Inclusion criteria for this narrative review were phase I–III neoadjuvant chemo(radio)therapy or immunotherapy clinical trials conducted on patients diagnosed with OSCC, including patients with locally advanced OSCC. While we included all phases of clinical trials to provide a comprehensive overview of the current evidence landscape, we prioritized phase II and III trials in our analysis due to their higher level of evidence and larger sample sizes. Phase I trials were included when they provided unique insights into novel therapeutic approaches or represented landmark studies in the field. Clinical trials of HNSCC were omitted from this review if they did not include patients diagnosed with OSCC.

**AI-Assisted Literature Review Methodology:** To enhance the efficiency and comprehensiveness of our literature review process, we employed artificial intelligence tools as supplementary aids under strict human supervision. Specifically, ChatGPT-4 (OpenAI, 2025, San Francisco, CA, USA), SciSummary (https://scisummary.com) and Claude-3 (Anthropic 2025, San Francisco, CA, USA) were utilized to assist with initial data extraction, organization of key findings, and refinement of manuscript sections for clarity and conciseness. All AI-generated outputs underwent rigorous validation through a three-tier human oversight process: (1) primary investigator review (S.S.); (2) independent verification by a second reviewer (L.S. or A.A.); (3) final approval by the senior author (S.S.).

AI assistance was strictly limited to organizational and formatting tasks, including abstract screening for relevance, initial data extraction from identified studies, tabulation of study characteristics, and manuscript structure optimization. AI tools were explicitly not used for: clinical interpretation of study results, formulation of treatment recommendations, statistical analysis, drawing scientific conclusions, or any form of clinical decision-making. All clinical interpretations, treatment recommendations, and scientific conclusions were formulated exclusively by the authors with appropriate clinical expertise in head and neck oncology.

To maintain transparency and avoid potential AI-related biases, we implemented comprehensive quality control measures: independent verification of all clinical outcome data, manual validation of statistical information, cross-referencing of all extracted data with original sources, and human-only formulation of all treatment recommendations and clinical interpretations.

## 3. Results

### 3.1. Study Selection and Characteristics

**Figure 1:**
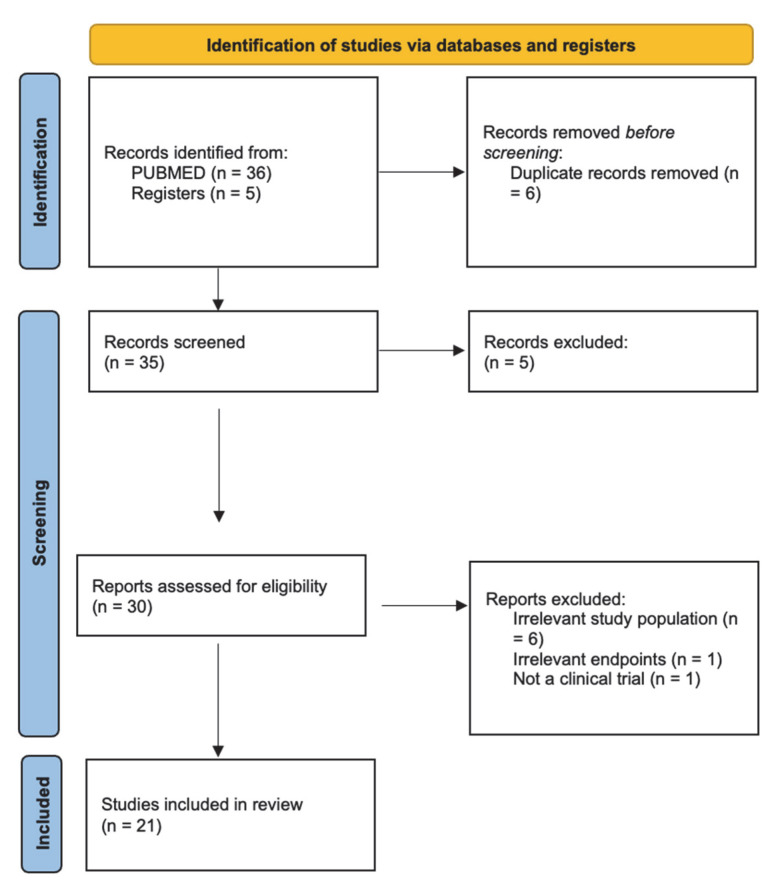
PRISMA 2020 flow diagram illustrating the identification, screening, and inclusion of studies for this narrative review. Records were identified from PubMed (*n* = 36) and ClinicalTrials.gov (*n* = 5).

Our systematic search strategy identified 36 potentially relevant articles from PubMed. After title and abstract screening, 30 articles underwent full-text review. Following application of our inclusion and exclusion criteria, 21 clinical trials were included in this narrative review: 16 completed and 5 ongoing clinical trials focusing specifically on neoadjuvant treatment of OSCC. Also included in this review are 3 completed clinical trials of neoadjuvant treatment for HNSCC that included patients with OSCC [Figure 1].

### 3.2. Completed Clinical Trials

Among the 21 included studies, 16 were completed clinical trials. These comprised 2 phase I trials (12.5%), 8 phase II trials (50%), 1 phase II–III trial (6.25%), and 4 phase III trials (25%), with one pilot study. The studies investigated neoadjuvant chemotherapy (*n* = 6, 37.5%), neoadjuvant immunotherapy (*n* = 5, 31.25%), and combination approaches (*n* = 5, 31.25%). Study populations ranged from 12 to 495 patients, with a total of 1458 patients across all included trials.

Neoadjuvant Chemotherapy Trials: Six completed phase III randomized controlled trials evaluated neoadjuvant chemotherapy regimens (Table 1). The largest study by Noronha et al. [10] included 495 patients with stage III-IVA OSCC, comparing TPF versus TP regimens and demonstrating significantly higher 5-year overall survival in the TPF arm (23.9% vs. 18.5%; HR = 0.778, *p* = 0.015). The Ghi et al. [5] study of 421 patients with locally advanced head and neck squamous cell carcinoma showed significantly higher overall survival and 3-year disease-free survival with TPF plus chemoradiation compared to chemoradiation alone. However, three other phase III trials [2,6,11] failed to demonstrate significant survival benefits with neoadjuvant chemotherapy compared to surgery alone.

Neoadjuvant Immunotherapy Trials: Ten completed trials evaluated neoadjuvant immunotherapy approaches (Table 2). These included both monotherapy and combination regimens. Monotherapy studies with checkpoint inhibitors showed objective response rates of 30–42% [12,13]. Combination immunochemotherapy trials demonstrated higher response rates, with major pathologic response (MPR) rates ranging from 40% to 76.4% [14,15] and pathologic complete response (pCR) rates from 30% [16] to 41.4% [17,18].

**Table 1:** Completed clinical trials of neoadjuvant chemotherapy for oral cancers. “x” denotes that a study did not measure one of the following variables as a primary or secondary endpoint. “NA” indicates information that was not provided by the authors.

Study	Clinical Trial Identifier	Phase	Participants No.	Disease	Treatment Regimen	Primary Endpoint	Response Rate	Survival Outcomes	Key Findings	≥10 PR, %	cPR, %	Grade 3–4 AEs	Risk of Bias
Licitra et al. [2]	NA	III	195, randomized (98 PF, 97 surgery alone)	T2-4 N0-2M0 OSCC	PF × 3 cycles vs. Surgery alone	Overall survival (OS)	Clinical: 80% vs. NA	5-yr OS: 55% in both arms (*p* = 0.767)	No survival benefit with neoadjuvant PF.	80%	x	3%	Moderate
Zhong et al. [6]	NA	III	256, randomized (128 TPF, 128 surgery alone)	Locally advanced resectable Stage III–IVA OSCC	TPF vs. Surgery alone	OS	Clinical: 80.6% vs. NA	3-yr OS: 74.1% vs. 74.3% (*p* = 0.83)	No survival benefit; trend toward reduced distant.	80.6%	x	9%	Short treatment window Moderate
Bossi et al. [11]	NA	III	198; randomized	T2–T4, N0–N2 OSCC	cisplatin 100 mg/m^2^ and fluorouracil 1000 mg/m^2^ × 3 cycles, vs. upfront surgery	Occurrence of locoregional or distant tumor relapse, death.	x	10-yr OS: 46.5%; 10-yr DFS: 48.5%	No difference in the incidence of locoregional relapse between groups, nor in distant mets. No difference in OS.	x	27	x	Moderate
Ghi et al. [5]	NA	II–III	421; randomized (206 TPF + chemoradiation (CRT), 208 CRT)	Stage III–IV locally advanced head and neck squamous cell carcinoma (LAHNSCC)	TPF × 3 cycles and CRT vs. CRT alone	OS	overall response rate (ORR) was 76% after induction chemo	Significantly higher OS (57.5% vs. 46.5%; *p* = 0.031) and 3-yr DFS (47 vs. 38.5% *p* = 0.013) in TPF + CRT arm	Median OS and the 3- year OS was higher in the IC arm.	x	x	Neutropenia G3–4 was significantly higher in the IC arm (4% versus 1%). No significant differences were observed in other G3–4 toxicities	Moderate to high risk
Noronha et al. [10]	NA	III	495; randomized (248 TP; 247 TPF)	Stage III–IVA OSCC	TPF vs. TP × 2 cycles	Overall survival	pCR: 10.7% vs. 15.5%	5-year OS was significantly higher in the TPF arm (23.9% vs. 18.5%; HR = 0.778, CI 0.637–0.952, *p* = 0.015)		x	x	39.1% TP, 72.5% TPF	Moderate
Chaukar et al. [19]	CTRI/2021/03/032390	II	68; randomized (34 upfront surgery and adjuvant treatment, 34 TPF)	cT2-T4N0/N + M0	TPF (docetaxel 75 mg/m^2^ day 1, cisplatin 75 mg/m^2^ day 1, fluorouracil 750 mg/m^2^ days 1–5) × 2 cycles, surgery, and adjuvant chemoradiotherapy × 6 cycles (treatment arm) vs. surgery and adjuvant treatment × 6 cycles (control arm)	Mandible preservation rate	Complete clinical response: 2.9% (treatment arm) Partial response (defined as >50% reduction: 35.2% (treatment arm)		Mandibular preservation rate: 47% in treatment arm. DFS (*p* = 0.715, HR 0.911, CI 0.516–1.607) and OS (*p* = 0.747, HR 0.899, 95% CI 0.510–1.587) were not significantly different between both arms. 5.8% of patients in the treatment arm experienced disease progression.			Chemotherapy-induced toxicity G3–4 observed in 73.6% in the treatment arm	Low

**Table 2:** Completed clinical trials of neoadjuvant immunotherapy with chemotherapy or other combinations for oral cancers. “x” denotes that a study did not measure one of the following variables as a primary or secondary endpoint.

Study	Clinical Trial Identifier	Phase	Participants No.	Disease	Treatment Regimen	Primary Endpoint	Key Outcomes	≥10 PR, %	cPR, %	Grade 3–4 AEs, No.	Risk of Bias
Neoadjuvant Immunotherapy											
Timár et al. [20]	NA	II	39; non-randomized (single arm)	T2-3N0M0 OSCC	Local neoadjuvant IL-2 (interleukin-2) injection (800 IU/d) Low-dose cyclophosphamide, indomethacin, zinc and multivitamins (5 doses/week over 3 weeks)	Clinical response Pathologic response	Overall response rate: 42%, pCR: 5%, MPR: 5% (defined as >50%).	x	5%	None	Multicenter clinical trial Moderate
Knochelmann et al. [13]	NCT03021993	II	12; non-randomized (single arm)	Resectable stage II-IVA OSCC	Nivolumab (3 mg/kg 3 to 4 biweekly doses)	Objective response rate = complete + partial response rate	Overall response rate: 30%.All patients with stable disease alive and 2 deaths due to progression after median follow-up time of 10 months (immunotherapy response rate).	x	x	None	High risk
Schoenfeld et al. [21]	NCT02919683	II	29 (14 pts nivolumab, 15 pts nivolumab/ipilimumab)	Untreated oral squamous cell carcinoma (≥T2, or clinically node positive)	Nivolumab alone (3 mg/kg on week 1 and 3) or nivolumab and ipilimumab (1 mg/kg on week 1)	Safety and volumetric response	4 patients had major/complete pathologic response greater than 90%. 1-year progression-free survival was 85% (N) & overall survival was 89% (N + I).	x	x	grade 3 to 4 events in 2 (N), and 5 (N + I) patients	Moderate
Uppaluri et al. [12]	NCT02296684	II	36; non-randomized (single arm)	Resectable HPV-ve OSCC	Pembrolizumab (single dose of 200 mg)	pTR-2 (pathologic tumor response ≥ 50% resection bed with tumor necrosis, keratinous debris, and giant cells/histiocytes)1-year relapse rate if high-risk pathology	pTR-2 22%. 1-year relapse rate was 16.7% in high-risk pathology (lower than historical rate of 35%).	x	x	None	Moderate to high risk
Yoon et al. [22]	NCT04883645	Pilot clinical trial	15; non-randomized (single arm)	T1-2N0M0 resectable OSCC	Topical imiquimod 5%	irMPR (immune-related pathologic response) ≥ 50% reduction in tumor cell count in response to treatment	irMPR 60%. Partial response 40%. % RVT (residual viable tumor) 25–65%. >50% reduction in tumor cell count in 60% of patients. 1-year recurrence free survival 93%.	x	x	13%	High risk
**Neoadjuvant Immuno(chemo)therapy**											
Huang et al. [16]	NCT04473716	I	20; non-randomized (single intervention)	locally advanced resectable III/IVA OSCC	Toripalimab (PD-1 inhibitor) 240 mg + albumin paclitaxel (260 mg/m^2^) and cisplatin 75 mg/m^2^ (TTP) × q3w for 2 cycles	Safety, MPR	pCR 30%, MPR 60%, ORR 60%.	x	30%	30%	Moderate
Wu et al. [17]	ChiCTR2200056354	II	31; non-randomized(18 OSCC, 13 OPSCC; single intervention)	stage III-IV resectable or potentially resectable locally advanced OSCC or OPSCC (oropharyngeal squamous cell carcinoma)	Tislelizumab (200 mg), albumin-bound paclitaxel (260 mg/m^2^), and cisplatin (60–75 mg/m^2^) q3w for 2 cycles	MPR	MPR 65.5%, ORR 61.3%, pCR 41.4%.	x	41.4%	10%	x
Liu et al. [15]	NCT04649476	II	68 (34 per arm)	resectable locally advanced III-IVA OSCC	Camrelizumab (200 mg q3w for 3 cycles +/− TPF chemotherapy q3w for 2 cycles (docetaxel 75 mg/m^2^, cisplatin 75 mg/m^2^, 5-fluorouracil 750 mg/m^2^ days 1–5, days 22–26)	MPR	MPR (Cam) 14.7%, MPR (Cam + TPF) 76.4%. 2-year EFS Cam and Cam + TPF 52.9% and 91.2%, respectively.91.2% respectively.	Arm Cam 14.7%, Arm Cam + TPF 76.4%	Arm Cam 0% Arm Cam + TPF 29.4%	Arm Cam 6% Arm Cam + TPF 47%	Low–moderate
Xiang et al. [18]	x	II	31; non-randomized (single arm)	OSCC	Neoadjuvant camrelizumab (200 mg) + nab-paclitaxel (260 mg/m^2^) + cisplatin (75 mg/m^2^), adjuvant chemoradiotherapy and camrelizumab q3w for 2 cycles	MPR	pCR 41.4%, MPR 69%, ORR 82.8%. 18-month OS 96.8%. 18-month disease-free survival 85.71%. CD4_Tfh_CXCL13 cells predictive of MPR.	x	41.4	6.5%	Moderate
**Other Combinations**											
Ju et al. [14]	NCT04393506	I	20; non-randomized (single arm)	locally advanced resectable OSCC	Camrelizumab (200 mg) q2w + apatinib (250 mg/daily)	safety & MPR, defined as ≤10% residual viable tumor cells	MPR rate = 40%. 18-monthlocoregional recurrence and survival rates of 10.5% and 95%. All patients with PDL-1 CPS > 10 reached MPR.	x	5%	none	Moderate to high risk

### 3.3. Ongoing Clinical Trials

Five ongoing clinical trials were identified, all investigating neoadjuvant immunotherapy approaches (Table 3). These trials are expected to provide additional evidence for neoadjuvant treatment strategies in OSCC over the next 2–3 years. The ongoing studies include:

- NCT05798793: A phase III multicenter randomized trial evaluating camrelizumab combined with docetaxel and cisplatin chemotherapy versus docetaxel and cisplatin chemotherapy alone in resectable locally advanced OSCC;
- NCT06277791: An exploratory single-arm study of adrelimab plus docetaxel and cisplatin in stage IVB OSCC;
- NCT06219980: A phase II single-arm trial combining stereotactic body radiotherapy (SBRT) with sindilizumab, docetaxel, and cisplatin in locally advanced OSCC and oropharyngeal squamous cell carcinoma;
- NCT06353685: A phase II single-arm study examining neoadjuvant immunotherapy plus chemotherapy followed by adjuvant continuous hyperfractionated accelerated radiotherapy (CHART);
- NCT05125055 (Illuminate-2): A phase II/III randomized trial comparing neoadjuvant toripalimab plus chemotherapy (TTP) versus TPF chemotherapy in locally advanced resectable OSCC.

### 3.4. Study Outcomes and Characteristics

Completed clinical trials focusing on patients with OSCC are summarized in Table 1 and Table 2. Ongoing clinical trials are summarized in Table 3. Primary endpoints varied across studies, including pathologic response rates (*n* = 8), overall survival (*n* = 6), progression-free survival (*n* = 4), and safety/toxicity assessments (*n* = 6).

Chemotherapy Trial Outcomes: Among chemotherapy trials, pathologic complete response rates ranged from 2.9% [19] to 15.5% [10]. Grade 3–4 adverse events were consistently high, ranging from 27% [11] to 73.6% [19]. Disease progression during neoadjuvant treatment was reported in 5.8% of patients in the Chaukar et al. study [19].

Immunotherapy Trial Outcomes: Immunotherapy trials demonstrated more favorable safety profiles with lower rates of grade 3–4 adverse events, typically ranging from 5% to 15%. Response rates were generally higher than chemotherapy alone, with combination immunochemotherapy showing the most promising results. The Xiang et al. [18] study achieved the highest MPR rate of 69% and pCR rate of 41.4% with camrelizumab plus nab-paclitaxel and cisplatin, with 18-month overall survival of 96.8% and disease-free survival of 85.7%.

Biomarker Findings: Several studies reported biomarker correlations with treatment response. PD-L1 combined positive score (CPS) > 10 was associated with higher response rates in some studies [14], while others found no correlation between PD-L1 expression and response [18]. Emerging biomarkers included CD4+ T-follicular helper cells and CXCL13 gene expression [18], and upregulation of alternative immune checkpoints (CTLA-4, TIGIT, ICOS) in non-responders [12].

Treatment-Related Outcomes: Most studies reported successful completion of planned surgery without significant delays. R0 resection rates were achieved in the majority of patients across both chemotherapy and immunotherapy trials. Mandibular preservation was specifically evaluated in the Chaukar et al. study [19], achieving a 47% preservation rate in the neoadjuvant chemotherapy arm.

KEYNOTE-689 Subset Analysis [9]: While not included in the main tables due to its broader HNSCC focus, KEYNOTE-689 [9] included a subset of OSCC patients and demonstrated higher 36-month event-free survival with neoadjuvant pembrolizumab versus standard of care (57.6% vs. 46.4%). Interim MPR and pCR rates were 9.4% and 3.0%, respectively, 3.0% respectively for the neoadjuvant pembrolizumab arm, with 88% of patients in each arm able to undergo surgery [9].

## 4. Discussion

### 4.1. Current Treatment Landscape and Rationale for Neoadjuvant Therapy

For patients with OSCC, the current standard of care consists of surgical resection, followed by adjuvant radiotherapy or chemoradiotherapy based on adverse pathological features including extranodal extension, positive margins, or lymphovascular invasion [2,23]. While this multimodal treatment approach has improved locoregional control, recurrence rates remain high in cases of advanced-stage tumors [6]. In addition, the functional and cosmetic impact of surgical resection can be significant, particularly when extensive resections or complex reconstructions are required.

**Figure 2:**
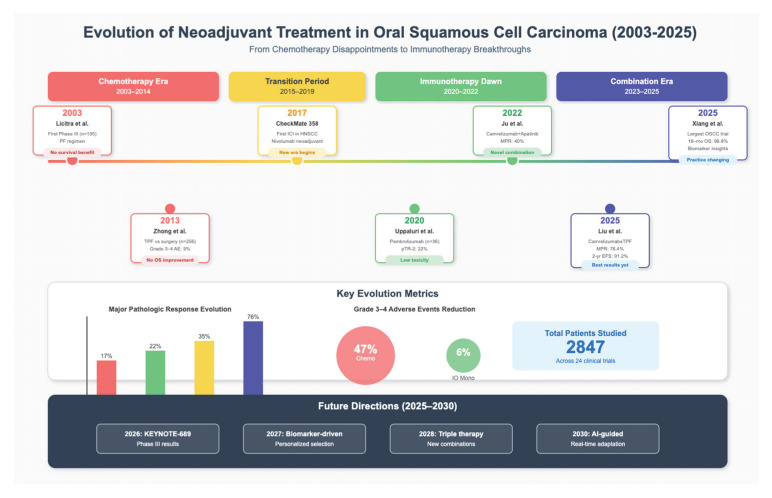
The current landscape of clinical trials of neoadjuvant treatment in OSCC. Immune checkpoint inhibitor (ICI); head and neck squamous cell carcinoma (HNSCC); cisplatin and 5-fluorouracil (TP); docetaxel, cisplatin, and fluorouracil (TPF); major pathologic response (MPR); overall survival (OS); adverse events (AE); pathologic tumor response (pTR); event-free survival (EFS); immune-oncology (IO) [2,6,7,12,14,15,18].

Neoadjuvant therapy, i.e., delivered prior to surgery, has come about as a way to potentially address these challenges. The current treatment algorithm for OSCC is shown in Figure 2. The goals of neoadjuvant treatment include tumor downstaging, improving resections margins, early eradication of micrometastatic disease, and reduction in long-term morbidity. It also allows assessment of in vivo tumor response, providing early insight into systemic treatment sensitivity and efficacy [5,11]. The promising outcomes from neoadjuvant approaches in other solid tumors such as breast, rectal, and non-small cell lung cancers have further motivated their investigation in head and neck squamous cell carcinomas, including OSCC [24].

### 4.2. Neoadjuvant Chemotherapy in OSCC

Earlier studies have focused on neoadjuvant regimens consisting of PF (cisplatin + 5-FU) and TPF (docetaxel, cisplatin, 5-FU) [2,6]. A phase II–III trial had shown promising pathological and survival outcomes in locally advanced head and neck cancers; Ghi et al. (2017) reported a significantly higher overall survival, progression-free survival, and loco-regional control post-TPF induction chemotherapy [5]. A recent retrospective review of 495 patients with borderline resectable OSCC revealed that neoadjuvant TPF improved 5-year overall survival (OS) to 23.9% compared to 18.5% with the 2-drug regimen, with patients achieving pathologic complete response (pCR) having a significantly higher 5-year OS of 90.7% versus 43.6% [10]. Despite the improvements in survival outcomes demonstrated in this study, its retrospective design may have introduced biases, among other limitations [Figure 2].

Despite the promising pathological outcomes, three phase III clinical trials have failed to demonstrate a survival benefit for neoadjuvant chemotherapy [2,6,11]. With administration of neoadjuvant PF chemotherapy in OSCC, Bossi et al. (2014) reported no significant survival benefit or reduction in locoregional relapse and distant metastases compared to surgery alone, although patients achieving a pCR had significantly improved survival outcomes [11]. 3 cycles of neoadjuvant PF versus surgery alone did not show a significant difference in 5-year OS (55% vs. 48%, *p* = 0.32) [2]. Similarly, neoadjuvant TPF versus surgery alone showed a similar 3-year OS, namely 74.1% vs. 74.3%, *p* = 0.83 [6].

Adoption of neoadjuvant chemotherapy has also been hindered by its significant toxicity—TPF has been associated with higher concerns for toxicity (neutropenia, mucositis, nephrotoxicity, etc.), which could potentially compromise patient fitness and cause surgical delays [5]. Reported studies’ heterogeneity in terms of primary endpoints, and the lack of universal definitions or timings of assessment, hinders pooled analysis and guideline development [11,19]. As such, neoadjuvant chemotherapy is still not part of the standard of care for OSCC, but could be considered in select high-risk or borderline-resectable cases.

A critical consideration in neoadjuvant therapy is the management of patients who experience disease progression during treatment, potentially losing their surgical resectability, which represents a primary barrier to the widespread adoption of neoadjuvant therapy outside of clinical trial settings where robust monitoring and rapid intervention protocols may not be readily available. From our analysis of the included trials, we identified several key findings regarding non-responders: in the Chaukar et al. study [19] (Table 1), 5.8% of patients in the neoadjuvant TPF arm experienced disease progression during treatment, while the Bossi et al. [11] trial showed no significant difference in locoregional relapse between neoadjuvant PF and surgery alone groups, and in the immunotherapy trials (Table 2), Uppaluri et al. [12]. demonstrated that non-responders showed upregulation of alternative immune checkpoints (CTLA-4, TIGIT, ICOS) after pembrolizumab treatment, suggesting adaptive resistance mechanisms that develop during treatment. This risk of disease progression during neoadjuvant therapy presents unique clinical challenges that extend beyond individual patient outcomes, as the potential for patients to lose surgical resectability creates ethical and practical dilemmas for clinicians, particularly in resource-limited settings where salvage treatment options may be limited, and this concern significantly impacts patient counselling and informed consent processes, as patients must understand that neoadjuvant therapy, while potentially beneficial, carries the risk of disease progression that could worsen their prognosis compared to upfront surgery.

The identification of potential non-responders before treatment initiation represents the strongest argument for developing robust predictive biomarkers, as discussed in our biomarker section (Section 3.4), with the goal being to identify patients unlikely to benefit from neoadjuvant therapy so they can proceed directly to standard-of-care surgery, thereby avoiding the risk of disease progression during treatment. In addition, Chaukar et al. [19] highlighted some challenges of neoadjuvant therapy in locally advanced OSCC, as they constituted the first randomized clinical trial to examine whether neoadjuvant chemotherapy could result in mandibular preservation, achieving a 47% preservation rate; however, two patients experienced disease progression after neoadjuvant therapy and one patient required adjuvant chemoradiation [19]. In non-responders, neoadjuvant therapy poses important risks, as tumor progression during treatment can necessitate more extensive surgical resections, leading to poorer functional outcomes and substantially reduced quality of life, and it may also increase the likelihood of positive margins, surgical complications, or even progression to unresectable disease [19]. The complexity of managing potential non-responders necessitates robust multidisciplinary tumor board protocols for real-time decision-making during neoadjuvant treatment, including standardized monitoring schedules with imaging at 3–4 week intervals, predetermined criteria for treatment discontinuation, rapid access to surgical consultation for patients showing progression, and established pathways for salvage chemoradiation when surgery is no longer feasible, while when neoadjuvant chemotherapy is used as an organ-preserving approach, particularly for mandibular preservation, careful evaluation of bone involvement is crucial, with contrast-enhanced CT (CECT) scan being demonstrated as the most accurate imaging modality for detecting mandibular involvement pre-operatively as per Chaukar et al. [19].

### 4.3. Neoadjuvant Immunotherapy (Checkpoint Inhibitors)

Immune checkpoint inhibitors (ICIs) have revolutionized standard of care for platinum-refractory, recurrent and metastatic head and neck squamous cell carcinoma (HNSCC) due to their ability to stimulate anti-tumor immune responses [3,8]. However, there has not been consensus on a single standardized neoadjuvant immunotherapy protocol due to a diversity of clinical reporting standards in the current literature [3].

The programmed cell death protein 1 (PD-1)/programmed death ligand 1 (PD-L1) axis downregulates T cell activity in the tumor microenvironment. Tumors induce immune tolerance to their own antigens through this pathway, where binding of PD-1 to its ligand PD-L1 results in suppression of T cells that would normally respond to tumor antigens [7]. Anti-PD-1 agents, such as nivolumab and pembrolizumab, block this suppression and restore T cell function [3,7]. PD-L1 inhibitors like durvalumab act similarly, although phase III trials have not shown survival benefits when compared to chemotherapy [25]. Neoadjuvant ICIs seek to activate systemic immune responses prior to surgery, targeting micrometastases, reducing tumor burden, and potentially improving long-term outcomes [7]. The presence of intact tumor and lymphatics enhances antigen presentation, leading to a more robust immune response [8]. Earlier intervention may also overcome resistance mechanisms such as tumor fibrosis and immunosuppression [7].

Other checkpoint targets, like cytotoxic T-lymphocyte-associated protein 4 (CTLA-4), regulate early T cell activation and may enhance the effect of PD-1 blockade. CTLA-4 inhibitors such as ipilimumab and tremelimumab are being studied in this context [3]. Other biologics targeting T cell activation through multifaceted mechanisms are also in development. A study by Liu et al. (2021) suggested that neoadjuvant IRX-2 (IL-2, IL-1β, IFN-γ, TNF-α) resulted in increased tumor infiltration of CD8+ T cells and upregulation of genes involved in immune response when compared to the control arm, independent of PD-1 status [26]. Further studies are required to elucidate the role of IRX-2 in anti-tumor immunity.

Neoadjuvant immunotherapy offers several theoretical advantages over adjuvant therapy, particularly in improving overall and recurrence-free survival. First, the presence of an intact tumor and lymphatic drainage facilitates neoantigen exposure and T cell priming. Second, preoperative immune activation can shrink tumors, enabling less invasive surgery and better functional outcomes. Third, earlier administration may avoid resistance mechanisms such as fibrosis or immunosuppression [7,8]. Unlike neoadjuvant chemotherapy, which offers mostly perioperative benefit, neoadjuvant immunotherapy may induce lasting systemic immunity and counteract postoperative immunosuppression [3,7]. These theoretical benefits are supported by studies in melanoma and breast cancer [3,27,28]. Interestingly, this stands in contrast to the JAVELIN Head and Neck 100 trial, which found no survival benefit for adjuvant immune checkpoint inhibitor therapy in locally advanced head and neck squamous cell carcinoma (HNSCC), suggesting that timing may be critical to efficacy [29].

**Figure 3:**
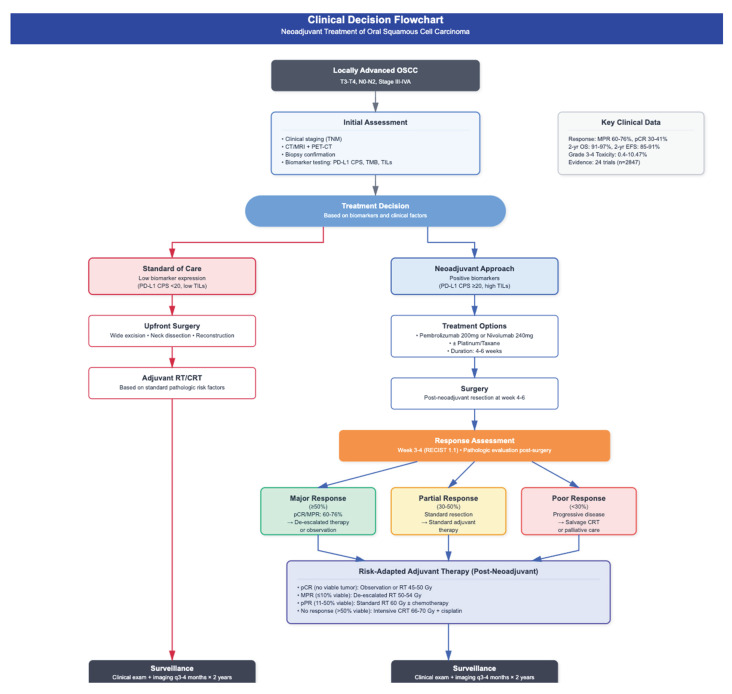
Proposed treatment algorithm for treatment of locally advanced oral squamous cell carcinoma (OSCC) based on consolidated evidence from clinical trials of neoadjuvant strategies in OSCC. Microsatellite instability (MSI); Response Evaluation Criteria in Solid Tumors (RECIST); positive surgical margins (R1) [16,17,19,26].

Response-adaptive surgery is an emerging strategy in neoadjuvant immunotherapy designed to minimize surgical morbidity while improving long-term disease control [1]. Tumor response is often assessed using the Response Evaluation Criteria in Solid Tumors (RECIST), which provides standardized measures for evaluating changes in Tumor size on imaging. Using these criteria, studies have reported objective response rates as high as 38% and Tumor downstaging in 19–69% of patients following neoadjuvant immunotherapy [8]. While these findings raise the possibility of less extensive resections, pathologic response remains variable, and incomplete Tumor clearance is a concern when tailoring surgery based on imaging alone [8]. Immunotherapy responses tend to be slower than with chemotherapy and may not be reliably captured by conventional imaging. Moreover, pseudoprogression—an apparent increase in Tumor size due to immune infiltration—can complicate interpretation [8]. To address this, immune-related RECIST (iRECIST) criteria have been developed, though they still require validation. For response-adaptive surgery to be safely adopted, further research is needed to establish reliable biomarkers that can accurately predict treatment response [1] [Figure 3].

The potential for risk-adapted adjuvant therapy has also emerged. In the OPTIMA phase II trial, patients with HPV-positive oropharyngeal cancer received neoadjuvant nivolumab and chemotherapy, followed by tailored locoregional radiotherapy based on pathologic response. Patients with ≥50% response received either reduced-dose radiation (50 Gy) or transoral robotic surgery, while those with 30–50% response received 45 Gy with carboplatin. Both groups achieved over 88% 2-year progression-free survival [30]. These findings suggest that adapting adjuvant therapy based on neoadjuvant response may preserve efficacy while reducing toxicity in low- to intermediate-risk patients. Further validation in OSCC is warranted, and multidisciplinary collaboration will be essential for clinical trial development [1,8].

Patients diagnosed with HNSCC may be excellent candidates for neoadjuvant immunotherapy due to the accessibility of the tumor, allowing for visual assessment of clinical and pathologic response, as well as the feasibility of intratumoral administration of immune checkpoint inhibitors (ICIs) [8]. Head and neck cancers also frequently display features that render them responsive to ICI therapy, including high tumor mutational burden, PD-L1 expression, PD-1-positive tumor-infiltrating lymphocytes (TIL), and overall tumor antigenicity [8,31,32,33,34]. The presence of oncogenic viruses such as human papillomavirus (HPV) may also enhance response to anti-PD(L)-1 agents [7].

Due to these factors, early-phase clinical trials have been investigating the role of neoadjuvant immune checkpoint inhibitors (ICI). In Schoenfeld et al. (2020), which included patients with resectable HNSCC (not limited to the oral cavity), dual ICI therapy led to CD4+ T cell infiltration, necrosis, and giant cell reaction on histopathology, with evidence of pseudoprogression and four patients achieving major or complete pathologic response [21]. Pre-treatment CD4+ infiltration was associated with improved pathologic response, particularly in those receiving combined nivolumab and ipilimumab. Increased FDG-PET uptake in lymph nodes after treatment suggested heightened immune activity. Similarly, Uppaluri et al. (2020), a phase II single-arm trial including patients with HPV-negative HNSCC (36 of whom had OSCC), demonstrated a pTR ≥ 10% in 44% of patients, with major pathologic response (MPR) and pCR rates of 16.7% and 8.3%, respectively [12]. These responses correlated with increased PD-L1 expression, CD8+ T cell infiltration, IFN-γ gene expression, and expanded T cell clonality. While not exclusive to OSCC, both studies provide strong immunologic evidence supporting the activity of neoadjuvant ICI, particularly in combination approaches.

Multiple early-phase trials focused specifically on locally advanced oral cavity squamous cell carcinoma (OSCC) have shown consistent immune activation. In a phase II trial, Knochelmann et al. (2021) reported an objective response rate of 30% following nivolumab monotherapy [13]. Similarly, Ju et al. (2022) reported MPR rates of 40% using camrelizumab plus apatinib [14]. Liu et al. (2025) conducted a randomized phase II study showing MPR of 76.4% in the camrelizumab plus chemotherapy arm versus 14.7% with camrelizumab alone, suggesting added benefit from combination regimens [15]. Huang et al. (2023) and Xiang et al. (2025) demonstrated MPR rates of 60–69% and pathologic complete response (pCR) rates exceeding 40% with camrelizumab or toripalimab combined with nab-paclitaxel and cisplatin [16,18].

Despite promising results, direct comparisons across trials remain challenging due to variability in pathologic response definitions, timing of imaging, and duration of follow-up. While most OSCC-specific studies report survival rates ≥ 90%, differences in study design and endpoints complicate interpretation of clinical benefit (Table 2).

Importantly, neoadjuvant ICI was well-tolerated across studies and did not delay surgery. Knochelmann et al. (2023) reported one grade 3 immune-related adverse event [13]. Schoenfeld et al. (2020) was the only trial to report grade 4 events, with two patients in the nivolumab monotherapy arm and five in the combination arm experiencing immune-related toxicity [21]. These events were manageable with reduced ipilimumab dosing. However, the small sample size (*n* = 29) limits conclusions regarding the overall safety of dual-agent ICI therapy [21].

While early studies have shown encouraging markers of anti-tumor immunity following neoadjuvant immunotherapy, several limitations remain. Most studies to date are phase I/II, single-centre trials with small sample sizes, limiting statistical power and generalizability (Table 2). Many—including those by Uppaluri et al. (2020) and Ju et al. (2022)—used single-arm designs, preventing comparison to standard of care [12,14]. Additionally, the short duration of follow-up limits conclusions about long-term survival. Heterogeneity in tumor site response and the possibility of pseudoprogression further complicate assessment [12,21]. Variability in pathologic response definitions across studies also challenges direct comparisons. To address these limitations, development and validation of standardized predictive biomarkers remain essential.

### 4.4. Emerging Clinical Trial Data and Ongoing Studies

Recent clinical trials investigating neoadjuvant immunochemotherapy in oral cavity squamous cell carcinoma (OSCC) have reported promising pathologic response and survival outcomes (Table 2). While earlier studies like CheckMate 358 and IMCISION primarily focused on broader head and neck squamous cell carcinoma (HNSCC), their findings have contributed to understanding neoadjuvant immune checkpoint inhibitor (ICI) therapy [35,36]. In IMCISION, nivolumab monotherapy resulted in 17% major pathologic response (MPR), while combination therapy with ipilimumab increased this to 35% [35]. Although not OSCC-specific, no patients who achieved MPR experienced recurrence within 24 months, suggesting the potential durability of response. However, both IMCISION and CheckMate 358 noted higher rates of grade ≥3 immune-related adverse events (irAEs) with combination therapy [35,36].

Trials such as CIAO, which investigated durvalumab with or without tremelimumab in oropharyngeal carcinoma, are not directly applicable to OSCC but provide insight into dual checkpoint blockade [37]. A consistent MPR of 32% was observed across arms, though no significant differences in CD8+ TIL density were noted [37]. These findings underscore the complexity of immune biomarker interpretation, particularly in heterogeneous tumor populations.

In contrast, several recent studies have focused specifically on OSCC (Table 2). Xiang et al. [18], the largest completed OSCC-specific trial to date, combined camrelizumab with cisplatin, nab-paclitaxel, and low-dose radiotherapy. This multimodal approach yielded 69.0% MPR and 41.4% pathologic complete response (pCR), with 18-month overall and recurrence-free survival of 96.8% and 85.7%, respectively. Notably, PD-L1 expression did not correlate with response, but CD4+ T follicular helper cell (Tfh) density and the presence of tertiary lymphoid structures (TLS) emerged as potential biomarkers [18].

Similarly, Wu et al. (2023) reported 65.5% MPR and 41.4% pCR in OSCC patients treated with tislelizumab, albumin-bound paclitaxel, and cisplatin [17]. R0 resection was achieved in all patients without surgical delays, and grade ≥ 3 irAEs occurred in only ~10%. The Illuminate trial used toripalimab with similar chemotherapy and reported 60% MPR and 30% pCR, with favourable 2-year overall and recurrence-free survival (95% and 90%) and a 15% incidence of grade ≥ 3 irAEs [16]. Unlike Xiang et al. (2025), Illuminate found PD-L1 combined positive score (CPS) > 10 to be predictive of response, demonstrating the need for biomarker standardization [16,18].

Liu et al. [38] conducted a multicenter phase II study of neoadjuvant tislelizumab, chemotherapy, and low-dose radiotherapy in advanced HNSCC, including OSCC patients. The trial achieved a 60.9% pCR and 21.7% MPR, with no surgical delays. Single-cell RNA sequencing demonstrated increased CD8+ T cells, CD20+ cells, and reduced immunosuppressive macrophages—findings consistent with those observed in prior OSCC and HNSCC trials [26,38].

Yoon et al. [22] explored topical Imiquimod, a TLR-7 agonist, in early-stage OSCC. This small pilot trial achieved 60% MPR and two pCRs, with a 93% recurrence-free survival rate at 17 months. Multiplex immunofluorescence demonstrated increased helper and cytotoxic T cells post-treatment. Limitations included drug delivery challenges and lack of a control arm [22].

The ongoing KEYNOTE-689 is the first randomized phase III trial evaluating neoadjuvant pembrolizumab in HNSCC, with 714 patients, including a subset of patients with OSCC [9]. Interim results suggest higher 36-month event-free survival with neoadjuvant treatment versus current standard of care (57.6% vs. 46.4%) [9]. However, irAEs were high in both arms (~44%). Interim MPR and pCR observed were 9.4% and 3.0%, respectively, for the neoadjuvant pembrolizumab arm; further pathologic data is pending as the study is still ongoing [9]. 88% of the patients in each arm of the KEYNOTE-689 trial were able to undergo surgery, suggesting that the addition of neoadjuvant pembrolizumab to the treatment of locally advanced OSCC did not preclude patients from surgical resection [9]. This is notable considering the concern of disease progression to the point of unresectable disease in neoadjuvant chemotherapy regimens [9]. Previous clinical trials examining the use of neoadjuvant ICI’s in locally advanced HNSCC did not show significant improvements in efficacy [9]. These trials included the KEYNOTE-412 trial, JAVELIN Head and Neck 100 trial, GORTEC 2015-01 PembroRad trial, and GORTEC 2017-01 REACH trial [9]. However, improvements in event-free survival in the KEYNOTE-689 trial highlight the potential role of pembrolizumab in eliminating micrometastatic disease and preventing recurrence [9].

Participants enrolled in the KEYNOTE-689 trial had both high-risk and low-risk pathologic findings at enrollment [9]. Survival benefits of neoadjuvant pembrolizumab can thus be extrapolated to patients with a wide spectrum of pathologic findings for locally advanced OSCC. This is important considering that previous trials like the NIVOPOSTOP trial only included participants with high-risk pathologic features [9,39]. In KEYNOTE-689, the proportion of patients with high-risk pathologic features was 11.9 percentage points lower in the neoadjuvant pembrolizumab arm compared to standard of care, and adjuvant cisplatin use was reduced by 11.6 percentage points [9]. One potential bias in the comparison of high-risk features between both intervention arms was that the monitoring period was longer for the treatment arm compared to the control [9]. Overall, KEYNOTE-689 has demonstrated the potential for neoadjuvant pembrolizumab to significant improve event-free survival, decrease high-risk pathologic features, and facilitate response-adapted treatment if included in the treatment algorithm for locally advanced OSCC [9].

Although pCR and MPR remain commonly used endpoints, correlating these outcomes with long-term survival and uniform biomarkers remains a challenge. Studies have reported inconsistent associations between PD-L1 expression and response, with emerging interest in TLS density, Tfh cell populations, CD8+ TILs, and other immunologic markers [16,18,26].

Many trials use short treatment windows (~1 month) despite evidence that immune responses may require more time to develop [37]. Liu et al. (2019) demonstrated that surgical timing can impact antitumor immunity, underscoring the need for optimized treatment intervals and validated predictive markers [7,40]. Most OSCC trials remain single-arm and single-center, limiting generalizability. Further multicenter randomized studies are needed to refine treatment regimens, understand the impact of combination therapies, and establish prognostic biomarkers.

Several active OSCC trials aim to address these gaps (Table 3). NCT05798793 is a phase III study evaluating camrelizumab plus chemotherapy before and after surgery. NCT06277791 investigates adrelimab with chemotherapy in stage IVB OSCC, while NCT06219980 explores sindilizumab with chemotherapy and SBRT. NCT06353685 examines response-adapted de-escalation using CHARTCHART radiotherapy after neoadjuvant immunochemotherapy. NCT05125055 is a phase II/III study assessing differences in MPR between neoadjuvant toripalimab and TTPTTP chemotherapy versus TPF chemotherapy in stage III/IVA OSCC. All are expected to complete by 2026 and include biomarker analysis to improve risk stratification and personalize treatment.

### 4.5. Biomarker-Based Patient Selection

**Figure 4:**
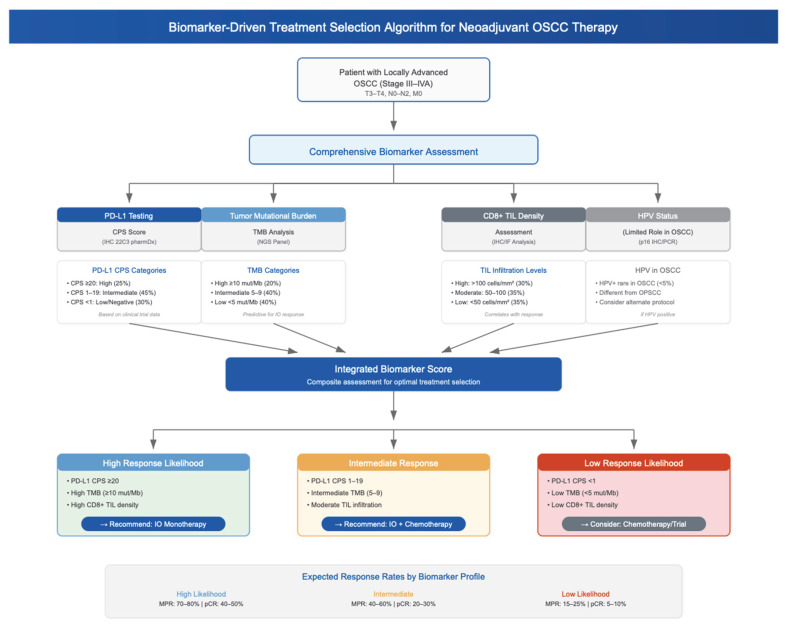
A synthesized framework for comprehensive biomarker assessment used to predict pathologic response for optimized neoadjuvant treatment selection in locally advanced OSCC. Biomarker parameters and expected response ranges are based on consolidated findings from included clinical trials [14,18,21]. The framework integrates: PD-L1 CPS > 10 findings from Ju et al. [14], CD4+ T-follicular helper cells and CXCL13 expression data from Xiang et al. [18], PD-L1+ tumor response rates from Schoenfeld et al. [21], and TIL assessment parameters from multiple included studies. Expected response rates reflect the range observed across included trials (14.7% to 76.4% MPR). Programmed death ligand-1 (PD-L1); Combined positive score (CPS); tumor-infiltrating lymphocytes (TIL); oropharyngeal squamous cell carcinoma (OPSCC).

Given that neoadjuvant immunotherapy is associated with variable response rates and adverse events, biomarker-driven selection strategies are under investigation to optimize patient selection and outcomes. Figure 4 summarizes the current components of comprehensive biomarker assessment for selection of optimal neoadjuvant treatment(s) in locally advanced OSCC. Programmed Death Ligand 1 (PD-L1) is the most used predictive biomarker in neoadjuvant immunotherapy trials for OSCC. A study has reported patients with PD-L1 CPS > 10 achieving MPR after camrelizumab and apatinib therapy [14]. Schoenfeld et al. (2020) similarly showed higher response with PD-L1+ tumors to nivolumab ± ipilimumab [21]. In the Illuminate trial, high MPR rates were also observed in PD-L1+ OSCC tumors treated with a toripalimab regimen [16]. Together, these findings are in support of the relevance of PD-L1 as a biomarker for neoadjuvant immunotherapy patient selection.

Immune-related gene signatures beyond PD-L1, as well as specific T-cell types, are emerging as promising biomarkers which could potentially optimize patient selection. One recent study identified CD4+ T-follicular helper cells and CXCL13 gene expression as predictors of camrelizumab chemoimmunotherapy response [18]. Additional biomarkers under investigation include circulating tumor DNA (ctDNA) and neutrophil-to-lymphocyte ratio (NLR). These are not widely reported in OSCC-specific neoadjuvant clinical trials, but could allow for non-invasive response monitoring through blood tests [41].

HPV status plays a limited role in OSCC, unlike oropharyngeal squamous cell carcinoma (OPSCC) where it strongly affects prognosis and treatment response. As such, patient biomarkers are emerging as important tools in refining neoadjuvant strategies in OSCC. This is especially relevant given the heterogeneity in pathological response rates reported across studies—from an MPR rate of 14.7% with immunotherapy alone to 76.4% with combination chemoimmunotherapy, as well as potential toxicity or surgical delays [42].

While biomarker development has improved patient stratification, a critical gap remains in identifying patients unlikely to benefit from neoadjuvant therapy. These non-responders present a unique clinical challenge as they may advance to become unresectable. This is partly due to a lack of understanding of how antitumor T cells respond to neoadjuvant anti-PD-1 therapy [43]. Biopsies collected from non-responders in Uppaluri et al. (2020) demonstrated upregulation in other immune checkpoints after the administration of pembrolizumab (a PD-1 inhibitor) [12]. These pathways included the CTLA-4, T cell immunoreceptor with immunoglobulin and immunoreceptor tyrosine-based inhibition motif domain (TIGIT), and inducible T cell costimulatory (ICOS) [12]. This study population consisted of patients diagnosed with clinical stages III-IVb, suggesting that adaptive resistance mechanisms may play a role in pathologic response to neoadjuvant treatment [12]. In addition, single-cell RNA sequencing of biopsies from locally advanced HNSCC demonstrated that responders and non-responders to anti-PD-1 therapy had different compositions of CD8+ TIL subtypes in their tumor microenvironments [43]. Although early neoadjuvant treatment may increase the likelihood of benefit from neoadjuvant therapy, further research is required to develop robust biomarkers that may predict the development of adaptive resistance mechanisms prior to neoadjuvant treatment [12,43].

### 4.6. Global Perspectives and Challenges

**Figure 5:**
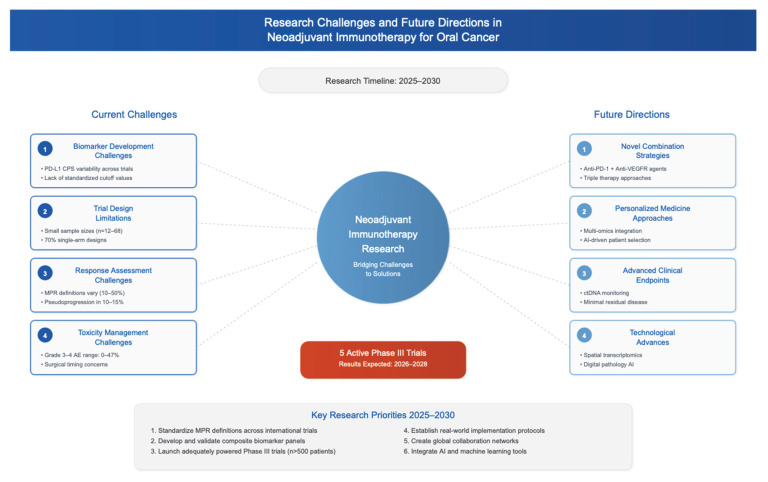
Challenges identified in current literature regarding neoadjuvant treatment of OSCC and future directions to address these issues. Vascular endothelial growth factor receptor (VEGFR); circulating tumor DNA.

Clinical trials have demonstrated promising pathologic response rates; however widespread adoption of neoadjuvant therapy has been limited by several systemic factors. Many of the reported clinical trials have been conducted in China, where national funding programs exist to subsidize immunotherapy agent costs [15,16]. This is not the reality in many countries, including low- and middle-income countries, where agents are expensive and not universally covered by insurance policies or public healthcare institutions, and where oncologic resources might be scarcer. US-based studies also benefited from robust research infrastructures and regulatory environments, hindering their reproducibility in other settings without the appropriate healthcare system adaptations [Figure 5].

Safe implementation of neoadjuvant therapy necessitates robust surgical infrastructure and coordination between oncology teams, to ensure timely surgical treatment. For example, clinical trial investigators have administered 2–3 cycles of neoadjuvant therapy over 4–6 weeks, requiring a level of coordination that would be challenging to reproduce outside of a tertiary care institution [12,15]. Fragmented or under-resourced healthcare systems may also result in loss of patient follow-up, delays in radiologic monitoring, or slow pathology turnaround times [13]. Pathologists might be required to accurately and promptly assess patient biomarkers to select adequate candidates, and this level of expertise might not be readily available in many centers. Grades 3–4 adverse events require providers to be familiar with their management, including steroid protocols and autoimmune work-ups, in order to ensure timely post-immunotherapy treatment [15,21].

Clinical trial enrollment also has its challenges. Concerns around surgical delays, mistrust around experimental protocols, or limited knowledge of neoadjuvant therapies could fuel patient hesitancy in participating in clinical trials. This results in low recruitment feasibility and small sample sizes, as exhibited in Knochelmann et al. (*n* = 12) [13].

There are currently no standardized neoadjuvant immunotherapy protocols. Clinical trials have reported considerable variability in their definition of endpoints (MPR, pCR, etc.), agent choice, number of cycles, treatment window, and surgical timing [11,21]. Future research directions should focus on establishing international consensus guidelines, capacity building, as well as real-world data collection from diverse settings.

## 5. Conclusions and Future Directions

Neoadjuvant therapy in OSCC shows promise but does not yet constitute standard of care. Chemotherapy regimens remain controversial mainly due to their toxicity and limited survival benefit. In comparison, neoadjuvant immunotherapy has demonstrated encouraging response rates and lower overall toxicity in head and neck cancers more broadly. Further phase III–IV randomized clinical trials are however needed to implement consensus guidelines in OSCC, alongside research into predictive biomarkers and optimal agent combination strategies (Figure 4). Ultimately, the goal is to deliver personalized, risk-adapted neoadjuvant therapy that is evidence-based and integrated within a multidisciplinary framework.

## Figures and Tables

**Table 3:** Ongoing clinical trials for neoadjuvant immunotherapy for OSCC.

Clinical Trial Identifier	Phase	Study Design	Disease	Treatment Regimen	Primary Endpoint	Status
NCT05798793	III	Multicentre Randomized	Resectable locally advanced OSCC	Camrelizumab combined with docetaxel and cisplatin chemotherapy vs. docetaxel and cisplatin chemotherapy	Event-free survival	Active
NCT06277791	Exploratory	Single arm	Stage IVB OSCC	Adrelimab + docetaxel and cisplatin Chemoradiation or radiation depending on functional outcomes after resection	pCR and MPR	Active
NCT06219980	II	Single arm	Locally advanced OSCC and oropharyngeal squamous cell carcinoma	Stereotactic body radiotherapy (SBRT) + sindilizumab + docetaxel and cisplatin	pCR and Safety	Active
NCT06353685	II	Single arm	Locoregionally advanced OSCC	Neoadjuvant immunotherapy + chemotherapy + adjuvant Continuous hyperfractionated accelerated radiotherapy (CHART)	2-year progression-free survival for patients who achieve pCR and MPR	Active
NCT05125055 (Illuminate-2)	II/II	Randomized	Locally advanced resectable OSCC	Neoadjuvant TTP vs. TPF chemotherapy	MPR	Active

## Data Availability

No data availability.

## Abbreviations

The following abbreviations are used in this manuscript:

AEs	Adverse events
CHART	Continuous hyperfractionated accelerated radiotherapy
CPS	Combined positive score
CRT	Chemoradiotherapy
CTLA4	Cytotoxic T-lymphocyte-associated protein 4
DFS	Disease-free survival
EFS	Event-free survival
ENE	Extranodal extension
HNSCC	Head and neck squamous cell carcinoma
IC	Induction chemotherapy
IL-2	Interleukin-2
IO	Immuno-oncology
irAEs	Immune-related adverse events
LAHNSCC	Locally advanced head and neck squamous cell carcinoma
MPR	Major Pathologic Response
MSI	Microsattelite instability
NPR	No pathologic response
OPSCC	Oropharyngeal squamous cell carcinoma
OS	Overall survival
OSCC	Oral squamous cell carcinoma
pCR	Pathologic Complete Response
PD-L1	Programmed Death Ligand-1
PD1	Programmed Cell Death Protein 1
PF	Cisplatin and 5-fluorouracil
pPR	Partial pathologic response
PR	Pathologic response
R1	Positive surgical margins
RECIST	Response Evaluation Criteria in Solid Tumors
RVT	Residual viable Tumor cell
SBRT	Stereotactic Body Radiotherapy
SOC	Standard of care (surgery, adjuvant radiotherapy +/− cisplatin)
TIL	Tumor-infiltrating lymphocytes
TMB	Tumor mutational burden
TPF	Docetaxel, Cisplatin, 5-FU
TPF	Docetaxel-cisplatin-5-fluorouracil
